# Reparative System Arising from CCR2(+) Monocyte Conversion Attenuates Neuroinflammation Following Ischemic Stroke

**DOI:** 10.1007/s12975-020-00878-x

**Published:** 2021-01-06

**Authors:** Joohyun Park, Jong Youl Kim, Yu Rim Kim, Meiying Huang, Ji Young Chang, A Young Sim, Hosung Jung, Won Taek Lee, Young-Min Hyun, Jong Eun Lee

**Affiliations:** 1 Department of Anatomy, Yonsei University College of Medicine, Seoul, Republic of Korea; 2 Brain Korea 21 Plus Project for Medical Science, Yonsei University College of Medicine, Seoul, Republic of Korea; 3 Brain Research Institute, Yonsei University College of Medicine, Seoul, Republic of Korea

**Keywords:** Monocytes conversion, Macrophages, CCR2, CX3CR1, Neuroinflammation ischemic stroke

## Abstract

Monocytes recruitment from the blood to inflamed tissues following ischemic stroke is an important immune response to wound healing and tissue repair. Mouse monocytes can be endogenously divided into two distinct populations: pro-inflammatory or classical monocytes that express CCR2^high^CX3CR1^low^ and circulate in blood, and anti-inflammatory or non-classical monocytes that express CCR2^low^CX3CR1^high^ and patrol locally. In this study of transgenic mice with functional CX3CR1^GFP/+^ or CX3CR1^GFP/+^-CCR2^RFP/+^, we found that CCR2^high^CX3CR1^low^ monocytes recruited to the injured brain were cytokine-dependently converted into CCR2^low^CX3CR1^high^ macrophages, especially under the influence of IL-4 and IL-13, thereby attenuating the neuroinflammation following sterile ischemic stroke. The overall data suggest that (1) the regulation of monocyte-switching is one of the ultimate reparative strategies in ischemic stroke, and (2) the adaptation of monocytes in a locally inflamed milieu is vital to alleviating the effects of ischemic stroke through innate immunity.

**Supplementary Information:**

The online version contains supplementary material available at 10.1007/s12975-020-00878-x.

## Introduction

Ischemic stroke, which causes devastating brain damage by reducing cerebral blood flow, is the second-leading cause of adult disability and death, after heart disease, worldwide [1, 2]. The inflammatory response following cerebral ischemia–reperfusion has become a rising issue in stroke research. Post-ischemic inflammation of the injured brain is characterized by the infiltration of blood immune cells and the interactions between resident microglia and invading blood immune cells [3–7].

There are heterogeneously two distinct subpopulations of monocytes, classical and alternative monocytes, that exist in murine species [8, 9]. Classical monocytes are actively recruited to inflamed tissues as they circulate through the blood. These monocytes are characterized by the expression of C-C chemokine receptor 2 (CCR2), which plays a pivotal role in homing to inflamed tissue. They also express high levels of lymphocyte antigen 6 complex locus C1 (Ly6C), low levels of C-X(3)-C motif chemokine receptor 1 (CX3CR1), and high levels of inflammatory cytokines and chemokines in inflamed tissue [2, 10–18]. These Ly6C^high^CCR2^high^CX3CR1^low^ monocytes subset is known as a short-lived and has pro-inflammatory and anti-microbial functions. In contrast, Ly6C^low^CCR2^low^CX3CR1^high^ subset is known as alternative or non-classical monocytes, which acts as an anti-inflammatory response and is characterized by CX3CR1-dependent recruitment to non-inflamed tissues and locally patrolling the vasculature [8, 19–21].

According to the previous research conducted up to date, these two populations of monocytes contribute to copious repair responses to the inflammation that follows various diseases. Although numerous studies have been executed on the subject of monocytes, little is known about the role of monocytes in ischemic stroke [22–25]. Therefore, to further investigate the precise role of monocytes, we used two-photon microscopy with two kinds of transgenic mice (CX3CR1^GFP/+^ and CCR2^RFP/+^-CX3CR1^GFP/+^) to delve into two subsets of monocytes dynamics during reparative processes after sterile cerebral ischemia. In this study, we have identified that pro-inflammatory CCR2^high^CX3CR1^low^ monocytes recruited to the ischemic injured brain were cytokine-dependently converted into anti-inflammatory CCR2^low^CX3CR1^high^ macrophages, overexpressing CX3CR1. Taken together, our overall data suggest that the regulation of locally secreted cytokines, which are related to the pro-inflammatory CCR2^low^CX3CR1^high^ monocyte conversion, is one of the potential therapeutic guides to alleviating the effects of cerebral ischemic milieu through innate immunity.

## Materials and Methods

### Animals

C57BL/6, 7 weeks old, male CX3CR1^GFP/GFP^, and CCR2^RFP/RFP^ mice were obtained from Jackson Laboratory. The generative protocols for the CX3CR1^GFP/GFP^ and CCR2^RFP/RFP^ mice were described previously [14, 26]. We generated C57BL/6, 12 weeks old, male CX3CR1^GFP/+^, CCR2^RFP/+^, and CX3CR1^GFP/+^-CCR2^RFP/+^ dual-reporter transgenic mice by mating the CX3CR1^GFP/GFP^ and CCR2^RFP/RFP^ mice with C57BL/6 wild type mice or each other. All animal experiments were approved by the Institutional Animal Care and Use Committee (IACUC) at Yonsei Laboratory Animal Research Center (YLARC). Animals were housed with food and water ad libitum and kept on a 12-h light/dark cycle.

### Transient Middle Cerebral Artery Occlusion

To induce focal cerebral ischemia, all mice were injected intraperitoneally (i.p.) with an appropriate mixture of zoletil (100 mg/kg, Virbac, USA) and xylazine (Rompun, 10 mg/kg, Bayer, Germany). The body temperature of the mice was maintained at 36.5–37.5 °C with a heating pad controlled by a rectal probe.

The animals were subjected to transient focal cerebral ischemia by intraluminal MCAO with a monofilament nylon suture, as previously described [27]. To summarize, the middle line neck of the mouse was incised, and the soft tissues were pulled apart. The right external carotid artery and the lower part of the right common carotid artery (rCCA) were ligated, and then the upper part of the rCCA was temporarily tied. A small hole was made between the upper and lower part of the rCCA. A 6-0 monofilament nylon suture with a head diameter of 200–210 μm was inserted into the right internal carotid artery to occlude the origin of the right middle cerebral artery (rMCA) in the circle of Willis. After 60 min of MCAO, blood flow was restored by withdrawing the suture, and regional cerebral blood flow was monitored using a laser Doppler flowmeter (Transonic Systems, Inc., Ithaca, NY, USA). For sham surgery, all physiological parameters were same as the mice of tMCAO group, but the 6-0 monofilament nylon suture was not introduced to into the rMCA.

### Immunohistochemistry

For the immunohistochemical examination, mice were sacrificed at 6 h, days 1, 3, or 7 after tMCAO. Mice (*n* = 5, each) were deeply anesthetized with an i.p. injection of a mixture of zoletil (100 mg/kg) and xylazine (Rompun, 10 mg/kg) and transcardially perfused with 4% paraformaldehyde in 0.1 mol/L phosphate-buffered saline (PBS). After cardiac perfusion, the brain was extracted and coronally cut into 2-mm thicknesses. The fixed brain tissues were washed with PBS for 6 h and placed in cryoprotectant (30% sucrose with 0.2% sodium azide) for 1 day. The tissue was embedded in OCT compound and transferred to − 80 °C. Frozen sections (*n* = 5, each) were sliced into 14-μm thicknesses and air-dried at room temperature. For the immunofluorescence analysis, the sections were fixed in methanol for 15 min at − 20 °C. After fixation, the sections were blocked with donkey serum (1:10, Abcam, Cambridge, UK) for 1 h at room temperature to prevent non-specific binding. The following primary antibodies were used overnight at 4 °C: rabbit monoclonal anti-Iba1 (1:200, Abcam, Cambridge, UK) and rabbit monoclonal anti-TMEM119 (1:500, Abcam, Cambridge, UK). The slides were washed in PBS three times and incubated with the appropriate secondary antibodies conjugated either with Alexa555 (1:200, Millipore, MA, USA) for 1 h at room temperature in a dark chamber. All images were acquired with a confocal microscope (LSM700, Carl Zeiss, Jena, Germany).

### Isolation of Microglia and Infiltrating Monocytes from the Brain

#### Single-Cell Dissociation from the Hemisphere

Mice (*n* = 5–6, each) were deeply anesthetized with a mixture of zoletil (100 mg/kg) and xylazine (Rompun, 10 mg/kg). After cardiac perfusion with cold PBS, the brain was removed, and the ipsilateral hemisphere was homogenized with a loose-fitting pestle in 15 mL of Dounce homogenizer containing 3 mL of RPMI to make a cell suspension. To further dissociate the tissues, they were passed through a nylon mesh (70 μm pore, SPL, Gyounggi-do, South Korea) in a 50-mL conical tube using the plunger of a 3-mL syringe, and RPMI was added to make the volume 7 mL.

#### Density Gradient

To isolate microglia and macrophages, stock isotonic Percoll (SIP) (9 parts Percoll stock: 1 part × 10 PBS) (PercollTM PLUS, GE Healthcare, Uppsala, Sweden) was made, and then 3 mL of SIP was added to the 7-mL cell suspensions in RPMI to make a final concentration of 30% SIP. The 10 mL of cell suspension was slowly layered on top of 70% SIP in a 15-mL polypropylene conical tube, which was then centrifuged for 30 min at 500 G and 18 °C. After centrifugation, the supernatant and debris were removed using a Pasteur pipette with a suction pump, and then 2–3 mL of the 70–30% junction was taken. Those cells were washed and centrifuged in a 15-mL conical tube with DPBS without CaCl2 and MgCl2 (WelGene, Gyeongsangbuk-do, South Korea) for 7 min at 500 G and 18 °C, and the process of washing and centrifuging was repeated three times. Finally, the pellet was resuspended with 1 mL of FACS staining buffer (BD Pharmigen™, New Jersey, USA) in a 1.5-mL tube and washed once using a micro-centrifuge at 10,000 G for 1 min at 4 °C.

### Flow Cytometry Analysis

All cells were stained with the following antibodies: FITC-conjugated CX3CR1 (clone SA011F11, BioLegend), Tmem119 (clone 106–6, Abcam), APC-Cy™7-conjugated CD11b (clone M1/70, BD Biosciences), and PE mouse anti-rabbit IgG (BD Biosciences). The incubation of all antibodies was performed for 15 min at 4 °C in the dark, and then centrifugation was carried out at 1500 rpm and 4 °C using a bench-top centrifuge. FACS staining buffer was used in the washing procedures of this experiment (BD Pharmigen™, New Jersey, USA). Afterwards, the cells were then filtered using a round-bottom tube with a cell strainer cap (STEMCELL Technologies). Cell data were acquired on an LSRII flow cytometer (BD Biosciences) and analyzed using FlowJo software (FlowJo LLC).

### Establishment of the Cranial Window Surgery for Two-Photon Intravital Imaging

The techniques used in the cranial window surgery have been described previously [28, 29]. In brief, to carry out two-photon microscopic intravital imaging of the brain, mice were deeply anesthetized by an i.p. injection of a mixture of zoletil (100 mg/kg) and xylazine (Rompun, 10 mg/kg). Each animal’s body temperature was maintained at 36.5–37.5 °C with a heating pad. For stable installation of the cranial window, mice were fixed in a stereotaxic frame (Live Cell Instrument, Seoul, Korea) during the procedure. A cranial window 4 mm in diameter was made with a dental drill in the right hemisphere, centered of 3 mm lateral to the midline and 1.95 mm posterior to bregma [30]. The exposed cerebral cortex was filled with 5 μL of saline, and the peri-region around the opened skull was fixed with cyanoacrylic glue. Then, the opened skull was covered with a 5-mm glass coverslip. The margin of the cranial window and skull area were filled with dental resin (B.J.M laboratory, Or-Yehuda, Israel) with a customized fixation ring. The fixation ring was then assembled with a stereotactic head fixation device (Live Cell Instrument, Seoul, Korea). After placement of a cranial window, all mice were injected with enrofloxacin (anti-biotic, Baytril, Bayer, Germany) and meloxicam (anti-inflammatory and analgesic drug, Metacam, Boehringer Ingelheim, USA) daily by an i.p and allowed to recover for additional 7 to 10 days before imaging to avoid any complications regarding neuro-inflammatory effects on imaging data [31].

### Intravital Imaging

For the intravital imaging, the mice (*n* = 3–5, each) were anesthetized with an i.p. injection of a mixture of zoletil (100 mg/kg) and xylazine (Rompun, 10 mg/kg). Their body temperature was maintained at 36.5–37.5 °C with a heating pad system (Live Cell Instrument, Seoul, Korea).

Texas red-conjugated dextran 70-kDa (Sigma-Aldrich) or CF®405M-conjugated wheat germ agglutinin (Biotium, CA, USA) was delivered via the retro-orbital sinus to visualize the brain vessel structure. For fine manipulation of the *X*–*Y*-axis, a staging system was set up using a Live Cell Instrument (LCI, Seoul, Korea). The animals were imaged using a two-photon microscope (LSM7MP, Zeiss, Germany), and the imaging data were computed using Zen software (Carl Zeiss, Germany).

Each imaged brain was excited with light at 820 nm or 880 nm for green, red, blue, and second harmonic generation. Images were acquired at a resolution of 512 × 512 pixels by a × 20 water-immersion objective lens. The imaging depth was 40–50 μm from the cerebral cortex surface with a *z*-stacking system of step sizes every 1 μm.

### Mouse Cytokine Assay

To evaluate the relative expression of cytokines and chemokines in the mice with cerebral ischemic injuries, a mouse cytokine array analysis was performed using mouse cytokine antibody array Panel A (ARY006, R&D Systems, Minneapolis, MN) according to the manufacturer’s instructions. In short, each brain hemisphere (*n* = 3, each) was extracted and homogenized to purify the proteins. The proteins extracted from the brain tissues were mixed with biotinylated detection antibodies and streptavidin-labeled horseradish peroxidase. The visualization was performed with a chemiluminescence-based detector, and the spot densities were quantified with HLImage ++ (Western Vision software, Salt Lake City, UT).

### Ex vivo Bioluminescent Measurement

To evaluate the intensity of EGFP- or RFP-expressing cells (monocytes and microglia that infiltrated the area after the ischemic stroke) over time, the mice (*n* = 5, each) were euthanized. The mice were deeply anesthetized through an i.p. injection of a mixture of zoletil and xylazine and then transcardially perfused with 4% paraformaldehyde in 0.1 mol/L PBS. After fixation, the brains were removed from the mice for further evaluation. Each extracted whole brain was placed on black paper to prevent autofluorescence in the background. Images of the region of interest were taken using an IVIS Spectrum (PerkinElmer, Waltham MA, USA).

### Isolation of Splenic Monocytes

#### Splenectomy

Splenectomies were performed using a protocol that was previously reported for the isolation of splenic monocytes/macrophages [32]. In brief, the mice (*n* = 5, each) were anesthetized with an i.p. injection of a mixture of zoletil (100 mg/kg) and xylazine (Rompun, 10 mg/kg), and an approximately 1-cm incision was made on the left side of the abdominal cavity under the ribs. The exposed spleen was removed by electrocauterizing the mesentery, connective tissues, and splenic vessels. All splenectomized mice were placed in CO2 chamber, and then euthanized.

#### Monocyte Harvest from the Spleen

To collect monocytes from the spleen, each extracted spleen was excised and minced using scissors and the plunger of 1 mL syringe in RPMI 1640 (Hyclone, Logan, UT, USA). The mixture was passed through a cell strainer (70 μm pore, SPL, Gyounggi-do, South Korea) for single-cell dissociation and then centrifuged at 1800 rpm for 10 min at 4 °C to isolate the splenocytes. To remove the red blood cells, the pellet was resuspended with 1 mL of ACK lysis buffer (Gibco, Massachusetts, USA) and incubated for 10 min at 37 °C. After incubation, the cells were centrifuged at 1800 rpm for 10 min at 4 °C and then process of washing was repeated 3 times with PBS. Finally, splenic monocytes were purified by negative selection using the EasySep™ mouse monocyte kit (Stemcell™ Technologies, Vancouver, Canada) according to the manufacturer’s instructions. After separation, the monocytes were re-suspended in a medium appropriate for the purpose.

### Monocyte Cultures

Monocytes harvested from the spleen were counted with Turk’s solution, suspended in RMPI 1640 supplemented with 10% FBS and a 1% penicillin (100 U/mL)–streptomycin (100 U/mL) mixture, and then plated at 4 × 10^5^ cells/mL in confocal dishes. Finally, cells were treated LPS (100 ng/mL) (O111:B4, *E. coli*, Merck), LPS with IL-4 (20 ng/mL) (Peprotech, London, UK), LPS with IL-13 (10 ng/mL) (Peprotech, London, UK), or LPS with IL-4 + IL-13 for specific time-points, respectively [33].

### Immunocytochemistry

Monocytes were fixed with 4% PFA for 10 min and then washed twice with PBS. After fixation, the cells were stained with DAPI for 5 min to counterstain them. After being washed twice with PBS, the samples were viewed under an LSM 700 confocal microscope (Carl Zeiss, Jena, Germany).

### Live Cell Imaging

Monocytes from the spleen purified by negative selection using the EasySep™ mouse monocyte kit (Stemcell™ Technologies, Vancouver, Canada) were stained with Hoechst 33342 for 10 min and washed 3 times with PBS. Then, they were re-suspended in phenol red-free RMPI 1640 medium (Thermo Fisher Scientific, Waltham, MA, USA) supplemented with 10% FBS and a 1% penicillin-streptomycin mixture and seeded at 4 × 10^5^ cells/mL in confocal dishes. For live imaging, monocytes were placed in an imaging chamber to maintain their temperature at 37 °C and control the humidity. Images were acquired using an epi-fluorescence microscope (Eclipse Ti2, Nikon, Japan), and the imaging data were computed with Volocity software (PerkinElmer, USA).

### Treatment of Blocking Antibodies

For IL-4 and IL-13 blocking experiments, mice (*n* = 3, each) subjected to tMCAO were injected intraperitoneally (i.p) 500 μg purified anti-IL-4 (clone 11B11, Sigma-Aldrich, USA) plus 500 μg purified anti-IL-13 (clone eBio13A, Invitrogen), or they were administered IgG1 i.p. 3 consecutive days after ischemic stroke.

### Measurement of the Infarct Size

After tMCAO, the mice were deeply anesthetized with a mixture of zoletil (100 mg/kg, Virbac, USA) and xylazine (Rompu, 10 mg/kg, Bayer, Germany). Brains (*n* = 3, each) were rapidly extracted and coronally sliced into 2-mm thick after cardiac perfusion with cold PBS, and the brain sections were transferred to a 2% 2,3,5-triphenyl-2H-tetrazolium chloride (TTC; Sigma-Aldrich, USA) and incubated at 37 °C for 20 min. The infarct size was measured by NIH ImageJ software and expressed as a percentage of the contralateral hemisphere to eliminate the contribution of edema to the calculation [34]. Finally, total infarct area was calculated by dividing the sum of infarct areas by the sum of contralateral hemisphere.

### Statistical Analysis

All statistical tests were performed using GraphPad software Prism version 6. All values were expressed as mean ± SD. Data were compared either by one-way ANOVA with a Bonferroni’s multiple comparison test or unpaired *t* test. Statistical significance was set at *p* < 0.05.

## Results

### The Number of Iba1^+^Tmem119^−^CX3CR1^GFP/+^ Cells Increased Days 3 and 7 After Ischemic Stroke

**Fig. 1:**
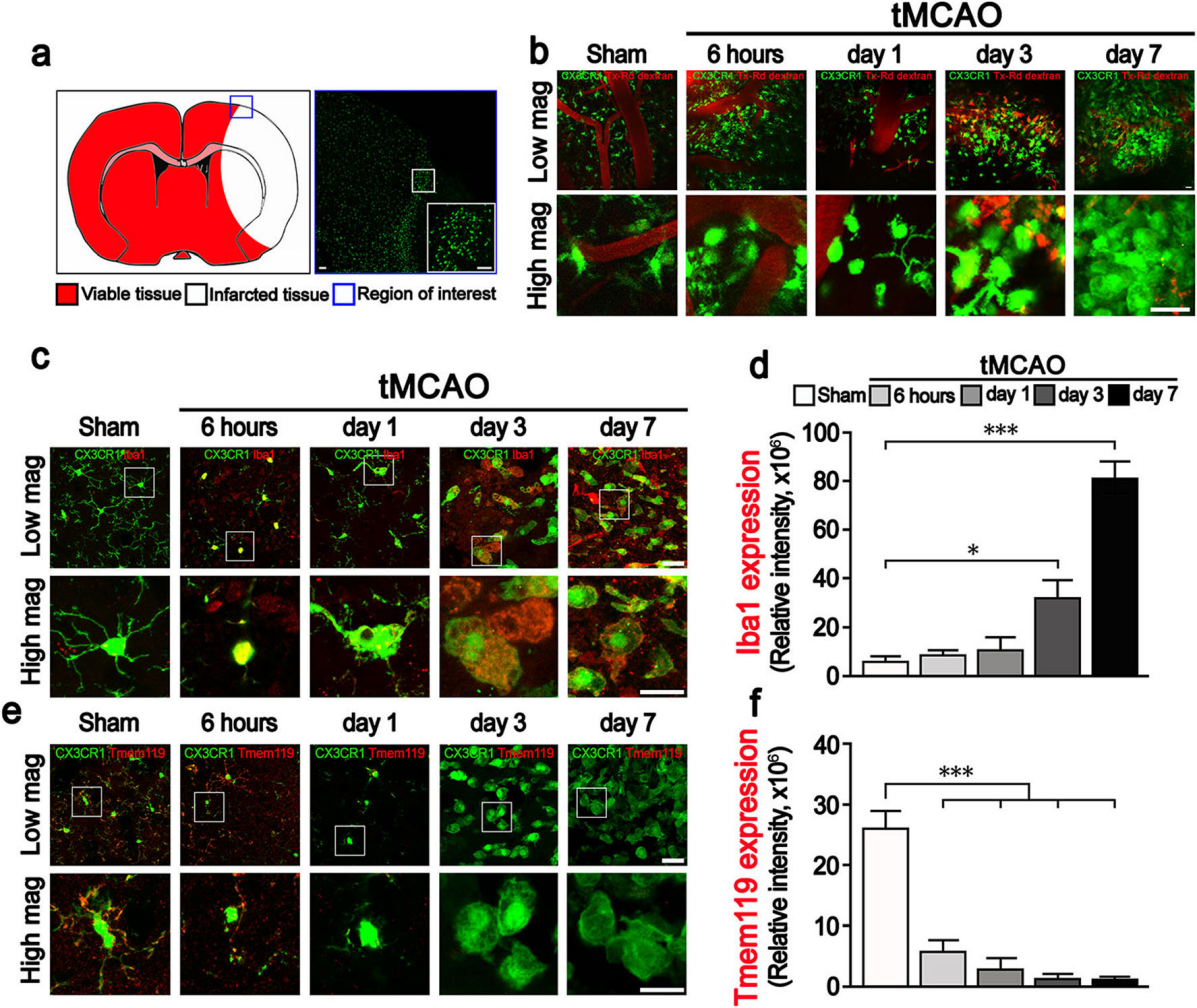
The Iba1^+^, Tmem119^−^, and CX3CR1^+^ cell levels were significantly elevated days 3 and 7 after ischemic stroke. **a** Illustrated TTC staining images of the ROI of the brain after ischemic stroke. Representative confocal images for ROI. Images were obtained from day 3 after ischemic stroke. Lens magnification, × 10 (tile scanning 10 × 10, blue box), × 30 (white box). Scale bar = 20 μm. **b** Captured images taken from sham to day 7 by two-photon microscope after ischemic stroke showing a gradual increase in CX3CR1(+) cells. High-magnification images of morphological alterations of GFP-expressing cells in a time-dependent manner. Blood vessels were labeled with Texas red dextran via the retro-orbital sinus. Scale bar = 20 μm. Lens magnification, × 20. **c, e** Images taken from sham to day 7 show a significant increase in Iba1, but a lack of Tmem119 expression. The expression of Iba1 and Tmem119 was detected by confocal microscope. Scale bar, 20 μm. Lens magnification, × 40. Data are representative of five independent experiments. **d**, **f** Quantitative analysis of relative fluorescence of Iba1- or Tmem119-expressing cells in a time-dependent manner after ischemic stroke. *n* = 5 mice per group independent experiments; error bars, mean ± SD (***p < 0.001, Bonferroni’s multiple comparison test)

To investigate histological changes in the microglia and blood-derived monocytes expressing CX3CR1 in brains with ischemic injuries, intravital imaging using two-photon microscope was performed in a time-dependent manner following ischemic stroke (Fig. 1a).

Two-photon intravital imaging showed that the number of GFP-expressing cells increased by days 3 and 7 compared with 6 h and 1 day after ischemic stroke. Interestingly, time-dependent morphological differences in GFP-expressing CX3CR1 cells were observed. At 6 h and 1 day after ischemic stroke onset, most observed cells had small cell bodies and ramified processes, whereas enlarged and ameboid cells were seen days 3 and 7 after ischemic stroke (Fig. 1b and Supplementary Movie 1-5). Also, these enlarged- and round-shaped GFP-expressing cells phagocytosed cell debris in the inflamed brain parenchyma day 7 after ischemic stroke (Supplementary Fig. 1). These results suggest two possibilities: (1) the increased number of enlarged cells originate from the resident microglia or (2) blood monocytes expressing CX3CR1 infiltrate into the injured brain and then differentiate into tissue macrophages.

Next, to confirm the results of the two-photon intravital imaging, immunohistochemistry (IHC) was conducted using the representative marker Iba1 for microglia and macrophages and Tmem119 as a specific marker for microglia. The immunohistochemical examination showed that Iba1 expression increased over time (% of relative intensity: 8.95 ± 2.72 at 6 h, 15.36 ± 1.94 at day 1, 32.49 ± 16.54 at day 3, 81.46 ± 13.10 at day 7) compared with the sham controls (4.97 ± 3.42) (Fig. 1c, d). The expression of Tmem119, however, decreased significantly in a time-dependent manner (% of relative intensity: 5.90 ± 3.99 at 6 h, 3.02 ± 2.97 at day 1, 1.44 ± 1.34 at day 3, 1.09 ± 1.41 at day 7) after ischemic stroke. In addition, the numerous CX3CR1^+^Iba1^−^, but CX3CR1^+^Tmem119^−^ cells found at days 3 and 7 were morphologically different from those of the sham group, and they were mostly observed in the peri-infarct site (Fig. 1e, f).

### The Increased GFP-expressing CX3CR1(+) Cells at the Site of Ischemic Injury are Blood Monocytes-derived Macrophages

**Fig. 2:**
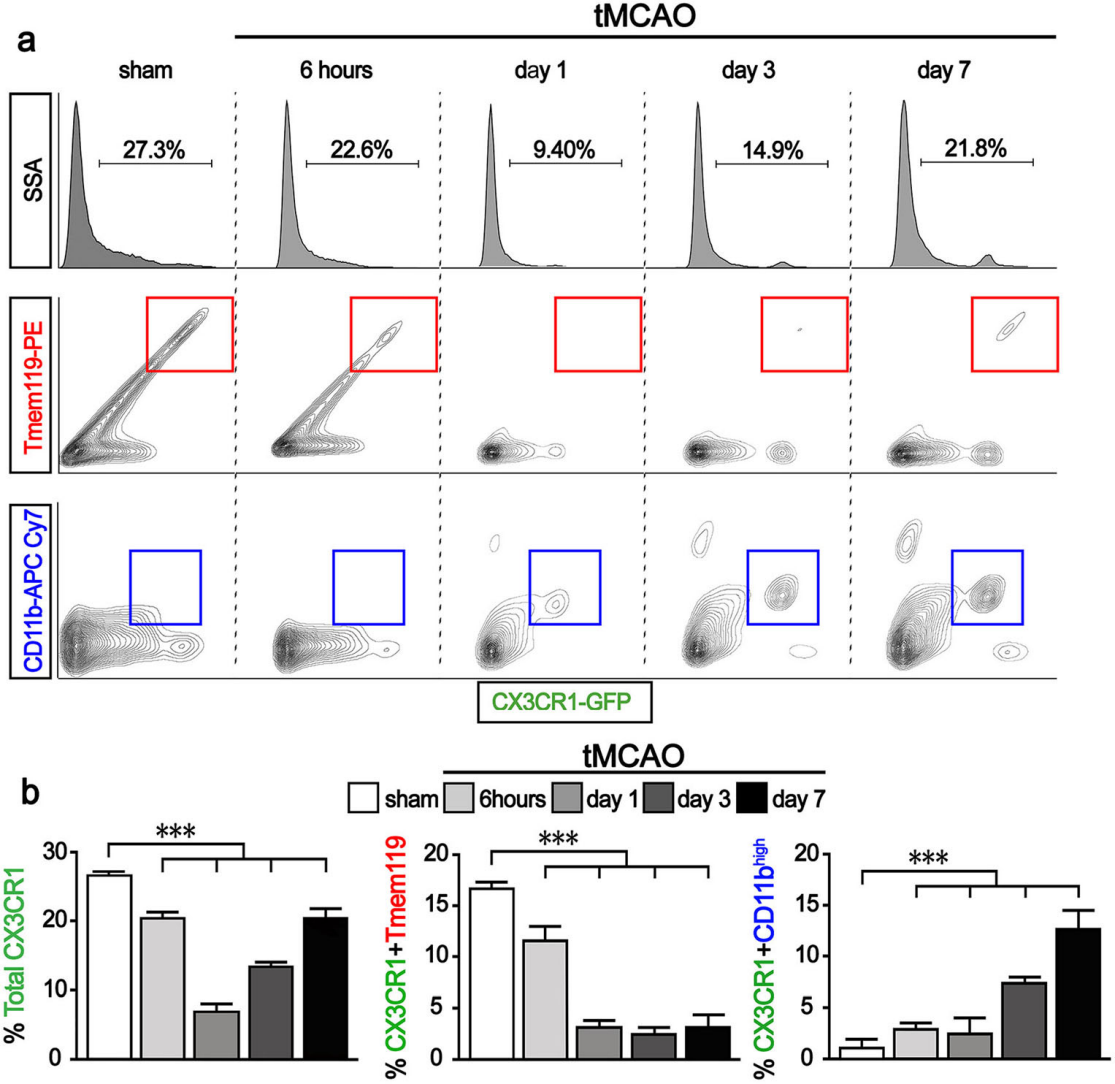
The increased GFP-expressing CX3CR1(+) cells come from the blood-derived macrophages. **a** Flow cytometry analysis for distinguishing blood-derived macrophages (CX3CR1^+^, Tmem119^−^, CD11b^high^) from microglia (CX3CR1^+^, Tmem119^+^, CD11b^high^). **b** Quantification of FACS analysis. Graphs shown are representative results from three different markers conjugated GFP, PE, and APC-CY, respectively. Data are presented as mean ± SD of at least five independent experiments (***p < 0.001, Bonferroni’s multiple comparison test)

To investigate proportional changes in microglia and monocytes after ischemic stroke, flow cytometry was conducted in CX3CR1^GFP/+^ mice. The relative percentage of total CX3CR1(+) cells from the brain decreased from 6 h (20.42 ± 2.64) to day 1 (6.87 ± 2.18) after ischemic stroke compared with the sham group (26.59 ± 1.37). On the contrary, the number of CX3CR1(+) cells increased significantly from day 3 (13.32 ± 1.55) to day 7 (20.33 ± 2.14) after ischemic stroke compared with the results from 6 h and day 1 (Fig. 2a). To confirm which cells, either proliferative microglia or blood monocytes-derived macrophages, compensated for the early loss of CX3CR1(+) cells, a further FACS analysis was conducted to distinguish between microglia and blood monocytes-derived macrophages using the anti-transmembrane 119 (Tmem119) and anti-CD11b antibodies. Tmem119 is a newly found transmembrane protein that is specifically expressed on microglia [35]. Also, with Tmem119, CD11b facilitates the distinction of myeloid lineage cells including monocytes and microglia from non-myeloid cells. As shown by those antibodies, the number of Tmem119 and CX3CR1-double positive cells decreased significantly over time (% of Tmem119 + CX3CR1: 11.59 ± 3.75 at 6 h, 3.17 ± 0.50 at day 1, 2.47 ± 0.21 at day 3, 3.14 ± 0.59 at day 7) after ischemic stroke compared with the sham group (16.68 ± 0.27). However, the number of CD11b^high^ and CX3CR1-double positive cells increased over time after ischemic stroke (% of CD11b^high^ + CX3CR1: 2.88 ± 0.09 at 6 h, 2.43 ± 0.56 at day 1, 7.35 ± 0.94 at day 3, 12.66 ± 2.10 at day 7) (Fig. 2b). Those observations indicate that the increased CX3CR1(+) cells on days 3 and 7 after ischemic stroke originated from infiltrating blood monocytes.

### Early-Invading RFP-expressing CCR2(+) Monocytes to Injured Brain Tissues was Changed into GFP-Expressing CX3CR1(+) Macrophages Days 3 and 7 After Ischemic Stroke

**Fig. 3:**
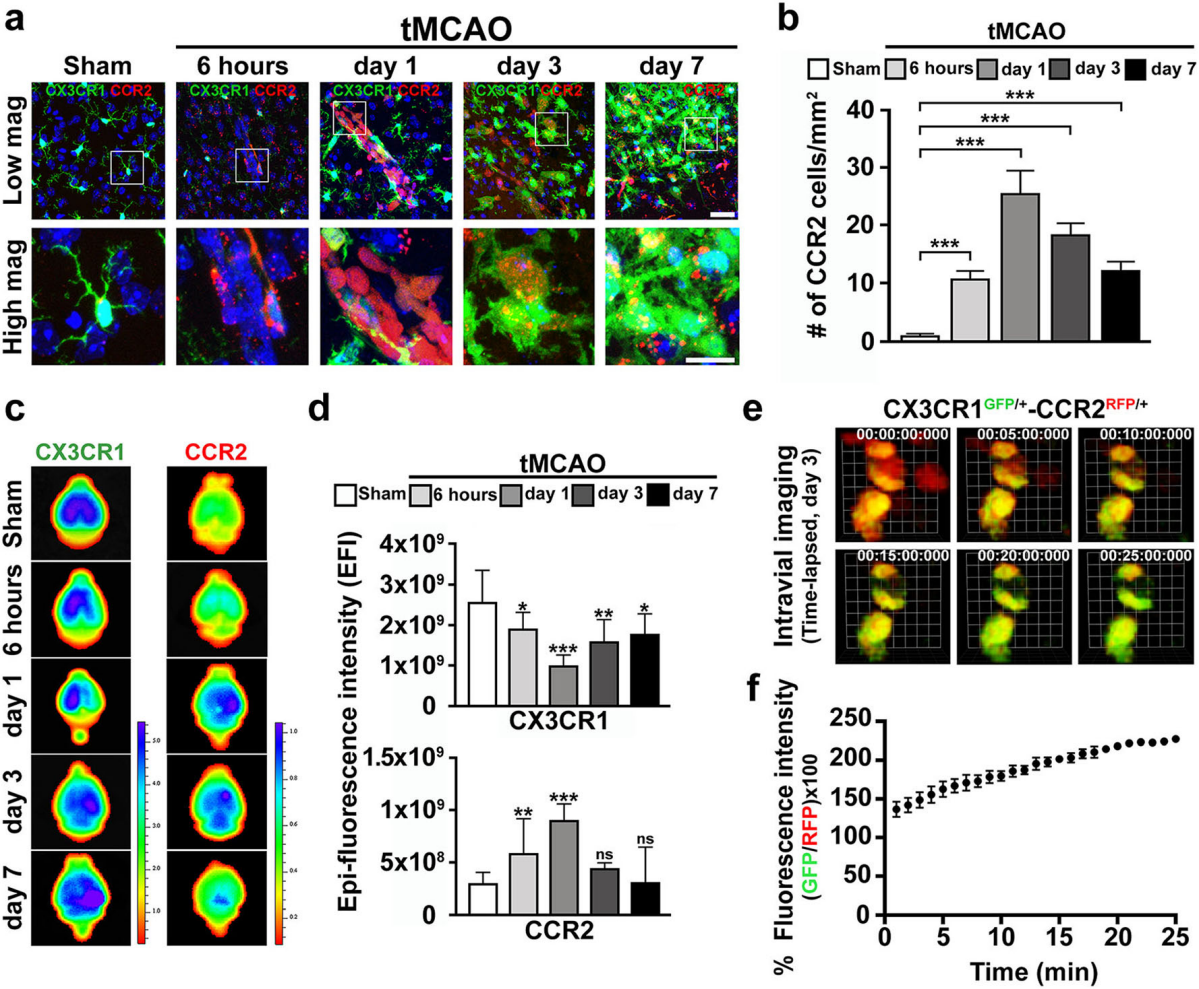
Infiltrating CCR2^high^CX3CR1^low^ blood-derived monocytes convert into CCR2^low^CX3CR1^high^ macrophages days 3 and 7 after ischemic stroke. **a** Immunofluorescent images showing CCR2(+) monocytes (red) located in the peri-infarctlesion of ischemic stroke using CX3CR1^gfp/+^-CCR2^rfp/+^ double-labeled functional transgenic mice. At days 3 and 7, CCR2(+) monocytes overexpress GFP, affecting morphological phenotypes. Scale bar = 20 μm. Lens magnification, × 40. **b** Quantitative analysis of infiltrating CCR2(+) cells. Data represent the mean ± SD of at least five independent experiments (*p < 0.05, **p < 0.01, ***p < 0.001, Bonferroni’s multiple comparison test). **c** Representative ex vivo brain images showing temporal changes in GFP or RFP epi-fluorescent intensity after ischemic stroke using CX3CR1^GFP/+^-CCR2^RFP/+^ double-labeled functional mice. **d** Quantitative analysis of fluorescence intensity in a time-dependent manner after ischemic stroke. Data represent the mean ± SD of five independent experiments (*p < 0.05, **p < 0.01, ***p < 0.001, Bonferroni’s multiple comparison test). **e** Time-lased intravital captured images showing the conversion of CCR2^RFP/+^ monocytes to CX3CR1-overexpressing macrophages day 3 after ischemic stroke. Scale bar (1 unit) = 5.3 μm. Lens magnification, × 20. **f** Quantitative analysis of fluorescence intensity of GFP and RFP over time after ischemic stroke

As described above, at least two distinct subsets of monocytes are reminiscent of macrophage phenotypes: classical/pro-inflammatory CCR2^high^CX3CR1^low^Ly-6C^high^-expressing monocytes and non-classical/alternative CCR2^low^CX3CR1^high^Ly-6C^low^-expressing monocytes [21, 36, 37]. We showed that numerous CX3CR1^GFP/+^ monocytes infiltrated the brain and were recruited from the blood to the inflamed tissue days 3 and 7 after the stroke in CX3CR1^GFP/+^ transgenic mouse. However, it was unclear whether these increased CX3CR1^GFP/+^ monocytes arose from CCR2^high^CX3CR1^low^ monocytes switching to CCR2^low^CX3CR1^high^ macrophages or from CCR2^low^CX3CR1^high^ monocytes themselves recruited after a delay. To determine it, we generated CX3CR1^GFP/+^-CCR2^RFP/+^ heterogenous dual-reporter transgenic mice and used them for time-dependent IHC experiments to observe any changes in the monocytes infiltrating the injured site. Those data show that CCR2^high^CX3CR1^low^ monocytes were recruited to the injured tissue from 6 h to day 1 (# of cells/mm^2^: 11.50 ± 1.38 at 6 h, 25.78 ± 3.87 at day 1) and peaked day 3 (18.29 ± 2.14) after ischemic injury compared with the sham group (0.71 ± 0.49). Also, the number of recruited CCR2^high^CX3CR1^low^ monocytes decreased at day 7 (# of cells/mm^2^: 12.71 ± 1.80) compared with the sham group. Interestingly, whereas the CCR2^high^CX3CR1^low^ monocytes (reddish) can be seen 6 h and day 1 after ischemic stroke, yellowish monocytes with colocalized GFP and RFP can be observed mainly days 3 and 7 after ischemic stroke. (Fig. 3a, b). These data suggest that the expression of GFP increased in a low to high fashion in the originally recruited CCR2^high^CX3CR1^low^ monocytes, thereby changing them into CCR2^low^CX3CR1^high^ macrophages after ischemic stroke. In addition to support our hypothesis and extend the results that imply the time-dependent transition of CCR2^high^CX3CR1^low^ monocytes into CCR2^low^CX3CR1^high^ macrophages after ischemic stroke, an ex vivo bioluminescent experiment was conducted. The results indicate that the radiant efficiency of GFP expression decreased at 6 h (epi-fluorescence intensity: 1.92e+09 ± 7.74e+08) and then further decreased by approximately 3-fold at day 1 (1.92e+09 ± 3.96e+08) following the deaths of numerous microglia after ischemic stroke, compared with the sham group (2.58e+09 ± 7.74e+08). However, GFP expression recovered by days 3 to 7 (1.60e+09 ± 5.32e+08 at day 3, 1.79e+09 ± 4.86e+08 at day 7) compared with that at 6 h and day 1 after ischemic injury. These data are similar to the FACS results. On the other hand, the RFP expression showed contradicting patterns compared to that of GFP; its intensity increased significantly and peaked day 1 (9.06e+08 ± 1.52e+08) after the acute phase of ischemic stroke. Then, RFP expression decreased, which led to no significances being found between the days 3 and 7 after ischemic brain injury group (epi-fluorescence intensity: 4.45e+08 ± 4.96e+07, 1.87e+08 ± 5.95e+07, respectively) and the sham group (3.02e+08 ± 1.02e+08) (Fig. 3c, d).

Next, to identify the CCR2^high^CX3CR1^low^ monocytes that switched to CCR2^low^CX3CR1^high^ macrophages in the peri-infarct lesion, two-photon intravital imaging was performed. Those data showed that under the basal condition, CCR2^high^CX3CR1^low^ monocytes are not present in brain parenchyma; instead, they circulate in the vasculature of tissues (Supplementary Movie 6). Early recruitment of CCR2^high^CX3CR1^low^ monocytes occurred 6 h after ischemic stroke (Supplementary Movie 7). Unlike the 6 h and day 1 (Supplementary Movie 8) data, CCR2^low^CX3CR1^high^ monocytes cells were abundantly present at days 3 and 7 after ischemic stroke (Supplementary Movie 9, 10). Similar to the immunohistochemical data as shown above, two-photon intravital imaging also indicated the invasion of CCR2^high^CX3CR1^low^ monocytes into injured brain tissues in the early stage of ischemic stroke. Interestingly, contrary to the results from 6 h and day 1, which show that CCR2^high^CX3CR1^low^ monocytes (reddish) were dominant after ischemic stroke, highly greenish CCR2^low^CX3CR1^high^ macrophages occupied most of the inflamed area on days 3 and 7 after ischemic stroke. In addition, time-lapse captured images showed that invading CCR2^high^CX3CR1^low^ monocytes convert into CCR2^low^CX3CR1^high^ macrophages day 3 after ischemic stroke. Also, the quantified imaging data show that percentages of GFP over RFP intensity (% GFP/RFP) increased days 3 after ischemic stroke (Fig. 3e, f and Supplementary Movie 11).

### Cytokines IL-4 and IL-13 Induce the Conversion of RFP-Expressing Pro-inflammatory CCR2(+) Monocytes into GFP-Expressing CX3CR1(+) Anti-inflammatory Macrophages Days 3 and 7

**Fig. 4:**
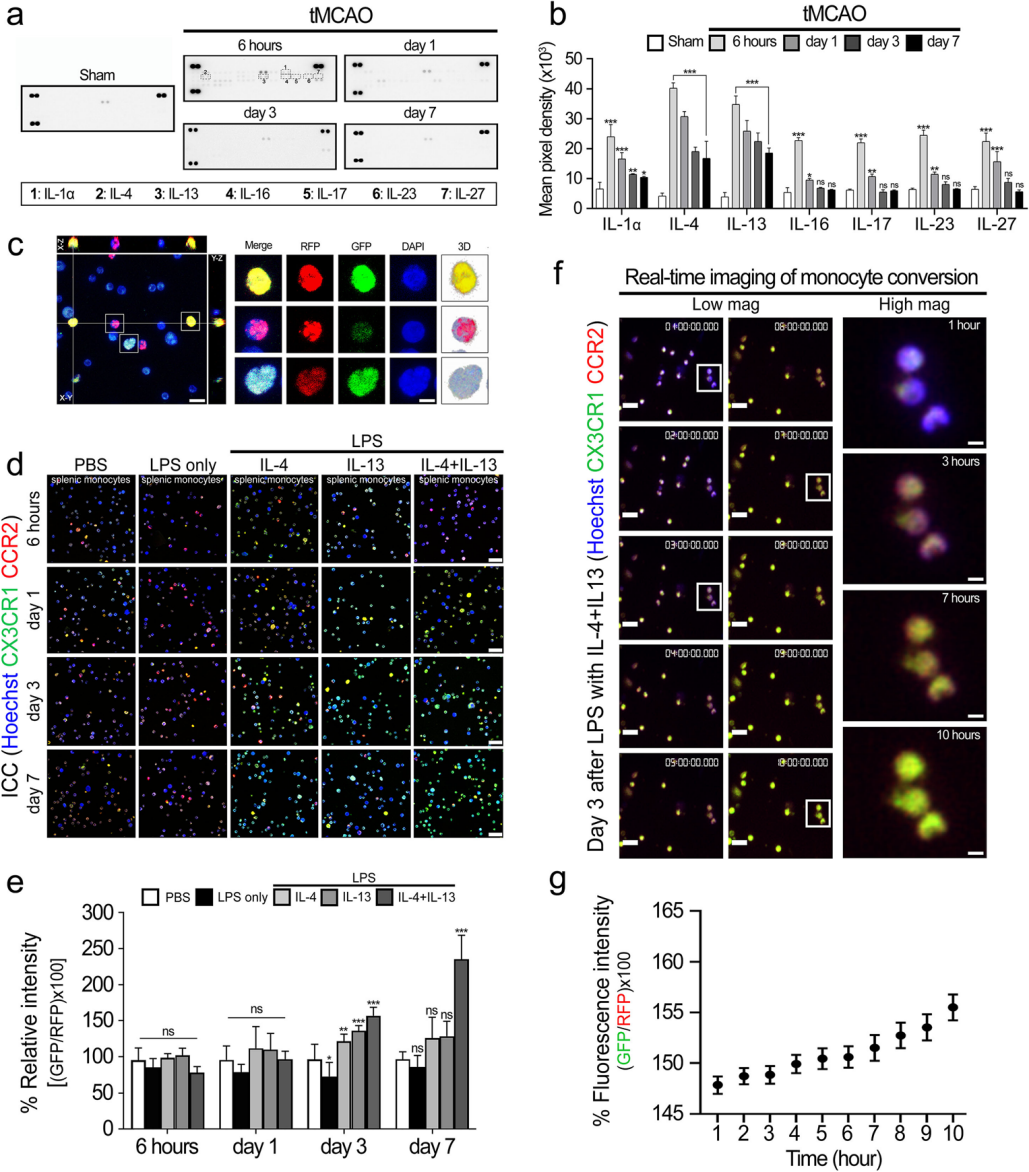
The induction of CCR2^high^CX3CR1^low^ monocytes switching into CCR2^low^CX3CR1^high^ macrophages by cytokines IL-4 and IL-13. **a** Representative images of protein expression profiles obtained by comprehensive protein array in each group of brain tissues. Black squares on a dotted line indicate representative cytokines. **b** Quantification of representative cytokine levels after ischemic stroke in a time-dependent manner. Data represent the mean ± SD of three independent experiments (*p < 0.05, **p < 0.01, ***p < 0.001, Bonferroni’s multiple comparison test). **c** Representative immunocytochemical images of conversion of CCR2^high^CX3CR1^low^ to CCR2^low^CX3CR1^high^ monocytes obtained by the confocal orthogonal projection technique after *z*-stack imaging. *X*-*Z* and *Y*-*Z* cross-sectional images show the co-localization of RFP and GFP. Comparative single-plane images of converted versus non-converted monocytes. Scale bar = 20 μm. Lens magnification, × 63. **d** Immunocytochemistry showing the conversion of isolated splenic monocytes. At days 3 to 7, the number of monocytes converted from CCR2^high^CX3CR1^low^ monocytes to CCR2^low^CX3CR1^high^ (yellowish color) was significantly increased by cytokines IL-4 and IL-13 when compared to sham and LPS groups. Scale bar = 20 μm. Lens magnification, × 40. **e** Quantitative analysis of the intensity of GFP- or RFP-expressing monocytes in a time-dependent manner. Data represent the mean ± SD of at least five independent experiments (*p < 0.05, **p < 0.01, ***p < 0.001, Bonferroni’s multiple comparison test). **f** Representative real-time images from a CCR2^RFP/+^-CX3R1^GFP/+^ double-labeled splenic monocytes. Scale bars = 20 μm. Lens magnification, × 20. Representative monocyte conversion images from white squares of low magnification images. Magnification, × 80. **g** Quantification of live imaging analysis. Graphs shown are representative results as mean ± SD of at least five independent experiments

To observe which cytokine affects the transition of monocytes, a cytokine array was conducted using an ipsilateral hemisphere of the brain after ischemic stroke in time-dependently. The results reveal that most cytokine levels were elevated, by 6 h after ischemic stroke, compared with the sham group. Elevated cytokine levels then decreased to day 7. However, IL-4 and IL-13 levels continued to be higher than those of the other cytokines on days 3 and 7 after ischemic stroke (Fig. 4a, b). Therefore, we selected those two cytokines to further investigate their effects for monocyte conversion.

To extend the cytokine array results and demonstrate that the conversion of CCR2^high^CX3CR1^low^ monocytes to CCR2^low^CX3CR1^high^ macrophages was specifically caused by cytokines IL-4 and IL-13, immunocytochemistry (ICC) using CX3CR1^GFP/+^-CCR2^RFP/+^ double-labeled monocytes isolated from spleens was conducted to confirm the GFP and RFP expression patterns over time.

The results show that more CCR2^low^CX3CR2^high^ macrophages are observed in LPS with IL-4 + IL-13–treated groups than in sham, LPS only, LPS + IL-4 or LPS + IL-13 single cytokine–treated groups on days 3 and 7. And the % relative intensities of GFP over RFP increased significantly in the LPS with IL-4 + IL-13 groups (157.0 ± 11.80 at day 3, 235.5 ± 33.2 at day 7) compared with the sham (96.5 ± 20.8 at day 3, 96.6 ± 10.2 at day 7) and the LPS only (72.7 ± 19.4 at 3 days, 85.7 ± 16.0 at day 7), LPS + IL-4 (121.5 ± 9.9 at day 3, 125.9 ± 29.0 at day 7), and LPS + IL-13–treated groups (136.2 ± 7.1 at day 3, 128.5 ± 20.8 at day 7) (Fig. 4d, e). Also, the immunocytochemical images obtained by the confocal orthogonal projection technique after *z*-stack imaging show the converted monocytes from CCR2^high^CX3CR1^low^ monocytes to CCR2^low^CX3CR1^high^ macrophages by cytokines IL-4 and IL-13 (Fig. 4c). In addition, to support the ICC confocal data and further investigation, we performed live cell imaging to acquire the point of conversion from CCR2^high^CX3CR1^low^ monocytes to CCR2^low^CX3CR1^high^ macrophages by IL-4 and IL-13 after LPS treatment. In those experiments, we confirmed that GFP expression started to increase from day 3 after treatment of IL-4 + IL-13 (Fig. 4f, g and Supplementary Movie 12). These results suggest that inflammatory monocytes expressing CCR2^high^CX3CR1^low^ could replace CCR2^low^CX3CR1^high^ macrophages through the influence of cytokines IL-4 and IL-13.

### Treatments with Anti-IL-4 and Anti-IL-13 Inhibits the Conversion of RFP-Expressing CCR2(+) Monocytes to GFP-Expressing CX3CR1(+) Macrophages and Infarct Reduction by Days 3 and 7 After Ischemic Stoke

**Fig. 5:**
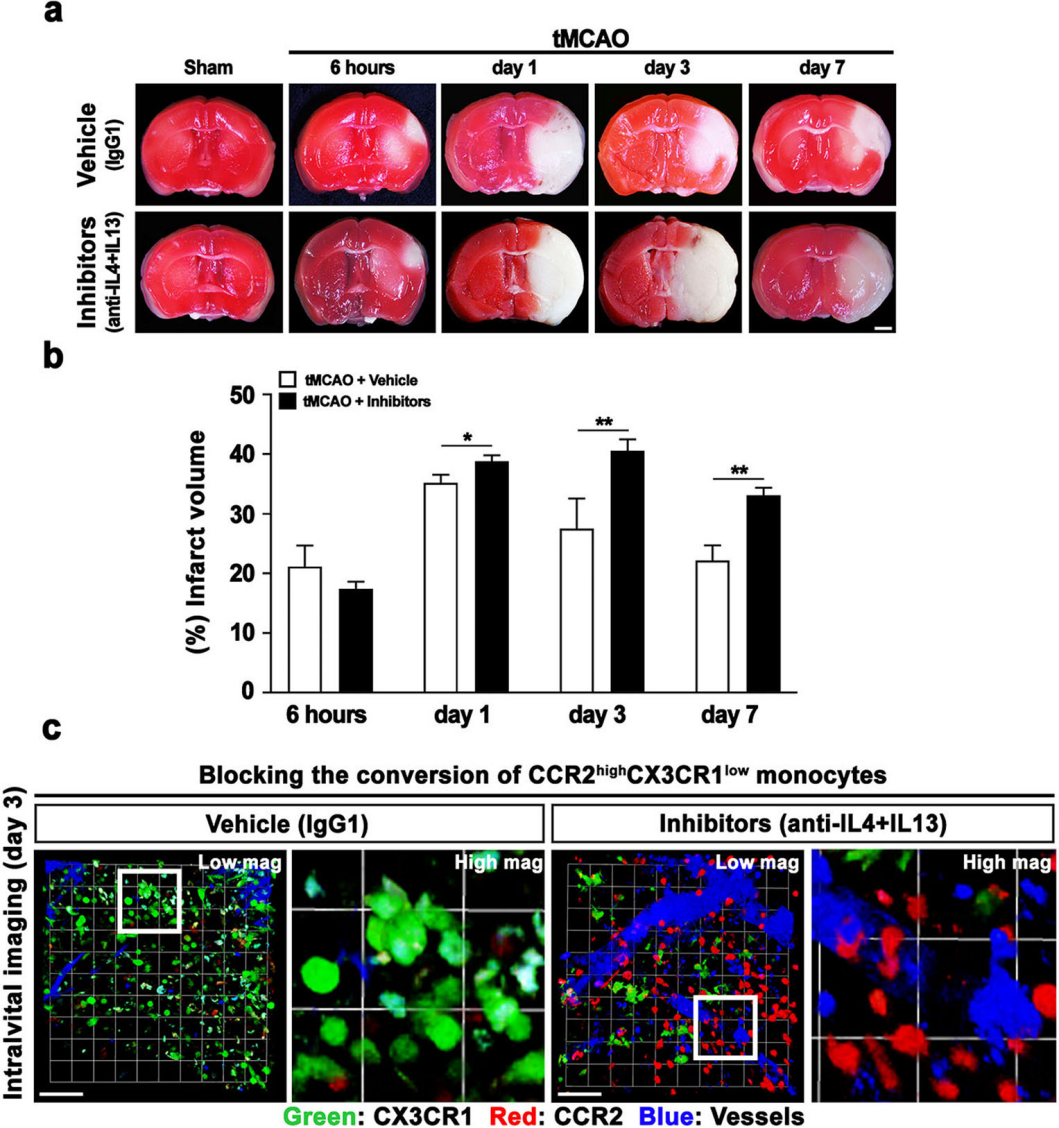
The treatment of IL-4 and IL-13 inhibitors affected the infarct volume and the CCR2(+) monocytes conversion into CX3CR1 macrophages after ischemic stroke. **a** Representative images showing the cerebral infarction by TTC staining, time-dependently. Scale bars = 2 mm. Lens magnification, × 4. **b** Quantitative analysis of cerebral infarct volume. Data are presented as mean ± SD of at least three independent experiments (*p < 0.05, **p < 0.01, unpaired *t* test). **c** Time-lapsed captured images obtained from intravital imaging showing the effects of inhibitors related with the CCR2(+) monocytes conversion (IgG1-treated vs Inhibitors-treated). Data are representative of three independent experiments. Scale bars = 20 μm. Lens magnification, × 20 (low mag.), × 64 (high mag.). Brain vessels shown in blue were stained with CF®405M-conjugated wheat germ agglutinin. CCR2 and CX3CR1 positive cells, which endogenously express RFP and GFP, were shown in red and green, respectively

To extend the cytokine array results and confirm the effects of IL-4 and IL-13 inhibitors, a time-dependent in vivo study was conducted. The results show that no significances were found between vehicle- and inhibitors-treated groups at 6 h (vehicle; 21.13 ± 3.53, inhibitors; 17.37 ± 1.22) and day 1 (vehicle; 35.20 ± 1.37, inhibitors; 38.73 ± 1.04) after ischemic stroke. However, % infarct volume was remarkably reduced in days 3 and 7 groups treated with anti-IL-4 and anti-IL-13 (46.53 ± 0.65 at day 3, and 36.27 ± 2.01 day 7) compared with the tMCAO only groups (27.53 ± 5.03 at day 3, and 22.17 ± 2.54 at day 7) (Fig. 5a, b). In addition, to verify the TTC staining data, we performed intravital imaging by two-photon microscope. Those results showed that RFP-expressing CCR2(+) monocytes were significantly increased in the group treated with anti-IL-4 and anti-IL-13 on day 3 after ischemic stroke compared with the IgG1 (vehicle)-treated group (Fig. 5c). These results suggest that cytokines IL-4 and IL-13 play an important role in stroke recovery and affect the conversion of CCR2(+) monocytes, but not the migration of CCR2(+) monocytes to the injured site after ischemic stroke. Additionally, to confirm our hypothesis, we performed magnetic resonance imaging (MRI) and IgG leakage staining to investigate correlations with vascular integrity determining the reason behind the increase in monocyte levels in the injured brain following ischemic stroke. Those results show that the cerebrovascular condition of the ipsilateral site was likely recovered by days 3 and 7 after ischemic stroke, compared with 6 h and 1 day after ischemic stroke (Supplementary Fig. 2 a). In addition, the percentage (%) of positive IgG area began increasing at 6 h (18.41 ± 5.25) and peaked at day 1 (38.25 ± 9.20). However, the IgG leakage area had decreased significantly by days 3 (13.36 ± 3.58) and 7 (4.57 ± 1.45) after ischemic stroke (Supplementary Fig. 2 b, c). These results indicate that large amounts of blood-derived CCR2(+) monocytes infiltrate the brain parenchyma at 6 h and day 1 following the blood–brain barrier disruption caused by ischemic stroke, and then the reparative process is initiated by locally secreted cytokine IL-4 and IL-13 in the ischemic milieu, thereby reprogramming the CCR2(+) monocytes to CX3CR1-overexpressing macrophages during days 3 and 7 after ischemic stroke.

## Discussion

The results of this study can be summarized as follows: (1) the increased CX3CR1(+) cells in injured brain tissues days 3 and 7 after ischemic stroke were infiltrating blood monocytes-derived macrophages. Moreover, it indicates that monocytes infiltrate into the damaged tissue at the early stage of ischemic stroke; (2) the monocytes mainly recruited to the inflamed brain tissues were CCR2^high^CX3CR1^low^ cells, by our specific time-point, they remained at least in the brain for 7 days after ischemic stroke; (3) those CCR2^high^CX3CR1^low^ monocytes converted into CCR2^low^CX3CR1^high^ macrophages day 3 after ischemic stroke by locally secreted cytokines IL-4 and IL-13; (4) the inhibition of cytokines IL-4 and IL-13 aggravates the infarct volume following ischemic stroke, thereby blocking the pro-inflammatory CCR2^high^CX3CR1^low^ monocytes conversion to CX3CR1-overexpressing anti-inflammatory CCR2^low^CX3CR1^high^ macrophages. Based on those results, we conclude that CCR2^high^CX3CR1^low^ monocytes infiltrate shortly after an ischemic stroke and are then affected by specific cytokines to overexpress CX3CR1, which converts them into CCR2^low^CX3CR1^high^ macrophages attenuating an ischemic stroke.

The primary focus of this study was to investigate the pathological roles of infiltrating monocytes in ischemic stroke. The well-organized model of transient focal cerebral ischemia, a sterile injury, is suitable for studying ischemic stroke and expounding the role of monocyte subsets. MCAO for 60 min produced a massive infarction causing neuronal death as well as the loss of glial cells including microglia in the MCA territory. This result differed from earlier reports of mild ischemic stroke, which led to delayed neuronal death, but not intact glial cells [38]. Differences were seen as the model of stroke used in this study is not a mild insult; while mild ischemic stroke produces delayed and selective neuronal death and leaves glial cells intact, including microglia, the massive ischemic strokes that were studied in this model led to the demise of neuronal cells as well as glial cells in the MCA lesion. In addition, a previous study already revealed the distinguishing features of macrophages derived from microglia and monocytes in mild episodic ischemic stroke [39]. Since there were copious studies regarding the interaction between resident microglia and invading monocytes, we specifically focused on the role of infiltrating monocytes in massive ischemic stroke. However, because we were concerned with infiltrating monocytes at specific time-points after ischemic stroke, our results might not be generalizable to all models of stroke. They do, however, reflect the characteristics and behavior of monocytes after a massive ischemic stroke. Extensive studies are still needed to determine the immunological and pathophysiological effects of ischemic stroke.

After ischemic stroke, the number of CX3CR1(+) cells increased on days 3 and 7 in the injured brain, and those cells were infiltrating blood monocytes–derived macrophages, not resident microglia, which we have established using transgenic mice (CX3CR1^GFP/+^). The specific markers used in this study, particularly Tmem119, can distinguish resident microglia from infiltrating monocytes, and those results were confirmed by flow cytometry and immunohistochemistry. To support the FACS and immunohistochemical data, we then showed that the increased number of CX3CR1^GFP/+^ cells at the injured brain parenchyma came from peripheral blood by using two-photon microscope after ischemic stroke. Those findings imply that the CCR2^low^CX3CR1^high^ macrophages were converted from the early-infiltrating CCR2^high^CX3CR1^low^ monocytes. There are some contradicting studies against our results reported that microglia proliferate and migrate to the injured site, accumulating at the border region of infarct, showing that the macrophages mainly populated in the peri-infarct lesion are microglia-derived cells after ischemic stroke [40, 41]. However, these require further investigation. In line with our hypothesis, Ritzel et al. revealed that microglia are vulnerable to severe ischemia. The study shows that the major population of increased cells in the ischemic injured lesion is infiltrating monocytes, early phagocytosing debris of dying cells, resulting in attenuation of ischemic stroke [42]. Also, Bennett et al. demonstrated that Tmem119 is a stable marker for resident microglia in peripheral injury, systemic inflammation induced by LPS, and traumatic CNS injury, indicating clear distinction between microglia and infiltrating monocytes [35]. That is why we chose the Tmem119 as an appropriate marker distinguishing microglia from infiltrating monocytes in the brain.

Using dual-labeled functional CCR2^RFP/+^-CX3CR1^GFP/+^ transgenic reporter mice, we found that most of the monocytes that early recruited to the inflamed brain tissues were classical monocytes and those monocytes started to differentiate and convert into CCR2^low^CX3CR1^high^ macrophages. Also, we verified that the CCR2^high^CX3CR1^low^ monocytes became CCR2^low^CX3CR1^high^ macrophages in the brain imaging by two-photon microscopy. In addition, we confirmed that real-time imaging of monocytes isolated from the spleen showed the CCR2(+) monocytes conversion by cytokines IL-4 and IL-13. We used splenic monocytes instead of blood monocytes because the yield of monocytes isolated from peripheral blood is extremely low (below 5%). However, when inflamed, the amount of monocytes in blood increases through the release of monocytes from the spleen [4, 12, 18, 36, 43–47].

As expected, the results from the cytokine array and immunocytochemistry show that the number of CCR2^high^CX3CR1^low^ monocytes becoming CCR2^low^CX3CR1^high^ macrophages increased more in the groups treated with LPS with IL-4 + IL-13 than in the sham, LPS only, LPS + IL-4, or LPS + IL-13 single cytokine–treated groups. Also, through MR imaging and IgG leakage staining, we identified that classical monocyte conversion could contribute to the repair process by decreasing IgG leakage and enhancing the vascular integrity in the cerebral parenchyma after ischemic stroke (data are not shown in the result part. See the supplementary 2). Moreover, in the real-time imaging, CCR2^high^CX3CR1^high^ monocytes conversion was greatly enhanced by overexpressing CX3CR1 day 3 after treatment with IL-4 and IL-13.

Today, there is a lot of controversy arising regarding the conversion of CCR2(+) monocytes. While there are studies that insist on sequential recruitment of CCR2^high^CX3CR1^low^ monocytes and CCR2^low^CX3CR1^high^ monocytes [48], rather than a conversion from one form to another, other studies indicate the latter, shown in different animal models and target organs [49–52]. In addition, a lately new paradigm challenging the central dogma of monocyte to macrophage differentiation has emerged. Jakubzick et al. revealed that classical monocytes were able to survey steady-state nonlymphoid tissues and transport antigen to lymph nodes, maintaining their markers and not differentiating into macrophages or dendritic cells [53]. This new concept of monocytes was further investigated in a study by Dal-Secco et al. In the study, Dal-Secco et al. demonstrated a transition from CCR2^high^CX3CR1^low^ to CCR2^low^CX3CR1^high^ cells within the tissue microenvironment and highlighted that the conversion of the recruited classical monocytes depended on a local milieu of cytokines IL-4 and IL-10 [25]. Indeed, previously reported data indicate that IL-4 or IL-13 plays pivotal roles for inducing the anti-inflammatory M2 phenotype of macrophages in various disease [45, 54–57]. These data support our hypothesis in that the transition of CCR2^high^CX3CR1^low^ monocytes into CCR2^high^CX3CR1^high^ macrophages is cytokine-driven. Although the monocyte-monocyte conversion by the reprogramming of classical monocytes was not found in the model of ischemic stroke, it is worth mentioning that (1) a cytokine-dependent transition from classical monocytes to reparative macrophages plays a vital role in temporal and spatial events for the endogenous restoration and (2) these macrophages mainly acting on neuroinflammation do not originate from resident microglia, but infiltrating blood circulating CCR2(+) monocytes following ischemic stroke.

Recently, Trzeciak et al. showed that CCR2(+) monocytes infiltrate the inflamed brain with significantly increased pro-inflammatory cytokine levels at the early phase of sepsis. Interestingly, these infiltrating monocytes induce the permanent microgliosis by self-proliferation of resident microglia in the cortex rather than converting themselves into anti-inflammatory macrophages, thereby attenuating the acute systemic inflammation of sepsis [58]. However, on the contrary of the Trzeciak’s results, in this study, the investigation was only confined to a model of sterile injury following ischemic stroke. Therefore, further studies are needed to determine whether similar responses are limited to the sterile injury models or occur in other tissues with non-sterile injury model. Furthermore, additional studies of the precise molecular mechanisms are necessary, in order to investigate the reason behind the statistical difference of the CCR2(+) monocytes conversion to alternative macrophages between the administration of both cytokine IL-4 and IL-13 and the single treatment of cytokine IL-4 or IL-13. Despite the results supporting the hypothesis through various experiments showing above, our study had several limitations. Although the two-photon microscopy is widely known to be suitable for intravital imaging that requires less phototoxicity, long-term imaging, and deep-tissue imaging compared with single photon confocal microscope, the possible imaging area is confined to the cortex of cerebral hemisphere, because the laser penetrating depth is maximally about 450 μm [59]. Moreover, the results by IVIS spectrum imaging are obtained from extraction of whole brain, meaning not the dynamic experiment of live animals, but post-mortem. IVIS is the equipment to measure the fluorescence on the surface of tissues called epi-fluorescence, however, which could result in false positives due to skulls and animal hair in living animals. That is the reason why we had no choice but to conduct in this way despite cognizing the fact that non-dynamic experiment of monocyte conversion; post-mortem analysis in time-dependent manners of each mouse.

Collectively, the present data demonstrate that monocytes are dynamically changeable depending on the microenvironment rather than existing in the dichotomy described previously. Furthermore, the CCR2^high^CX3CR1^low^ monocytes conversion in injured brain tissue could be a part of the anti-inflammatory response to the reparative process that helps to maintain homeostasis in the microenvironment after ischemic stroke. Our finding of the monocytes conversion in inflamed tissues could provide new insights into ischemic stroke. Targeting the regulation of monocyte conversion could be a novel and revolutionary approach to open the window in field of stroke treatment.

## Supplementary Information

ESM 1 (ZIP 1649 kb)

ESM 2 (ZIP 16347 kb)

## Data Availability

The datasets used and/or analyzed in the current study are available from the corresponding author upon reasonable request.

## Abbreviations

CCR2: C-C chemokine receptor 2; CX3CR1: C-X(3)-C receptor 1
Ly6C: lymphocyte antigen 6 complex locus C1
MCAO: middle cerebral artery occlusion
IL-4: interleukin 4
IL-13: interleukin 13
LPS: lipopolysaccharide
BBB: blood–brain barrier
PBS: phosphate-buffered saline
GFP: green fluorescence protein
RFP: red fluorescence protein
