# Supplementary material for: Reparative System Arising from CCR2(+) Monocyte Conversion Attenuates Neuroinflammation Following Ischemic Stroke
Source: Transl Stroke Res. 2021 Jan 6;12(5):879–93. doi: 10.1007/s12975-020-00878-x (PMC8421302; doi:10.1007/s12975-020-00878-x)

**Supplementary Information**

**Supplementary Figure 1. GFP-expressing CX3CR1 cells phagocytosed vessel debris day 7 after ischemic stroke.** Captured images obtained from intravital imaging showing the phagocytosis of enlarged CX3CR1^GFP/+^ cells. Scale bars = 20 μm. Lens magnification, 20x (low mag.), 64x (high mag.). Green: CX3CR1^GFP/+^ cells, Red: debris from brain vessels stained with Texas red-conjugated dextran.

**Supplementary Figure 2.** **The changes of vascular integrity after ischemic stroke.** **(a)** Data showing the MR angiography in time-dependent manners. **(b)** Images showing IgG leakage in the brain parenchyma after ischemic stroke. Scale bar = 20 μm. Lens magnification, 40x. Data are representative of five independent experiments. **(c)** Quantitative analysis of positive IgG leakage area. Data are presented as mean ± SEM of at least five independent experiments. (****p* < 0.001, Bonferroni’s multiple comparison test)

**Supplementary Movie 1.** Two-photon intravital imaging of the middle cerebral artery (MCA) region of brain (Sham). Scale bar = 50 μm, Lens magnification, 20X. Green: CX3CR1^GFP/+^, Red: brain vessels, Time stamp: hh/mm/ss.

**Supplementary Movie 2.** Two-photon intravital imaging of the middle cerebral artery (MCA) lesion 6 hours after ischemic stroke. Scale bar = 50 μm, Lens magnification, 20X. Green: CX3CR1^GFP/+^, Red: brain vessels

**Supplementary Movie 3.** Two-photon intravital imaging of the middle cerebral artery (MCA) lesion day 1 after ischemic stroke. Scale bar = 50 μm, Lens magnification, 20X. Green: CX3CR1^GFP/+^, Red: brain vessels

**Supplementary Movie 4.** Two-photon intravital imaging of the middle cerebral artery (MCA) lesion day 3 after ischemic stroke. Scale bar = 50 μm, Lens magnification, 20X. Green: CX3CR1^GFP/+^, Red: brain vessels

**Supplementary Movie 5.** Two-photon intravital imaging of the middle cerebral artery (MCA) lesion day 7 after ischemic stroke. Scale bar = 50 μm, Lens magnification, 20X. Green: CX3CR1^GFP/+^, Red: brain vessels

**Supplementary Movie 6.** Two-photon intravital imaging of the middle cerebral artery (MCA) region of brain (sham) using CX3CR1^GFP/+^-CCR2^RFP/+^ dual-reporter functional transgenic mouse. Scale bar = 50 μm, Lens magnification, 20X. Green: CX3CR1^GFP/+^, Red: CCR2^RFP/+^, Blue: brain vessels, Time stamp: hh/mm/ss.

**Supplementary Movie 7.** Two-photon intravital imaging of the middle cerebral artery (MCA) lesion 6 hours after ischemic stroke. Scale bar = 50 μm, Lens magnification, 20X. Green: CX3CR1^GFP/+^, Red: CCR2^RFP/+^, Blue: brain vessels

**Supplementary Movie 8.** Two-photon intravital imaging of the middle cerebral artery (MCA) lesion day 1 after ischemic stroke. Scale bar = 50 μm, Lens magnification, 20X. Green: CX3CR1^GFP/+^, Red: CCR2^RFP/+^, Blue: brain vessels

**Supplementary Movie 9.** Two-photon intravital imaging of the middle cerebral artery (MCA) lesion day 3 after ischemic stroke. Scale bar = 50 μm, Lens magnification, 20X. Green: CX3CR1^GFP/+^, Red: CCR2^RFP/+^, Blue: brain vessels

**Supplementary Movie 10.** Two-photon intravital imaging of the middle cerebral artery (MCA) lesion day 7 after ischemic stroke. Scale bar = 50 μm, Lens magnification, 20X. Green: CX3CR1^GFP/+^, Red: CCR2^RFP/+^, Blue: brain vessels

**Supplementary Movie 11**. Data representing 3D time-lapsed intravital imaging of CCR2(+) monocytes conversion. Scale bar = 5.2 μm/unit, Lens magnification, 20X, Green: CX3CR1^GFP/+^, Red: CCR2^RFP/+^

**Supplementary Movie 12**. Live imaging of splenic CCR2(+) monocytes conversion. Scale bar = 5.2 μm/unit, Lens magnification, 20X, Green: CX3CR1^GFP/+^, Red: CCR2^RFP/+^, Blue: nuclei stained with Hoecsht dye.

**Supplementary Figure 1**


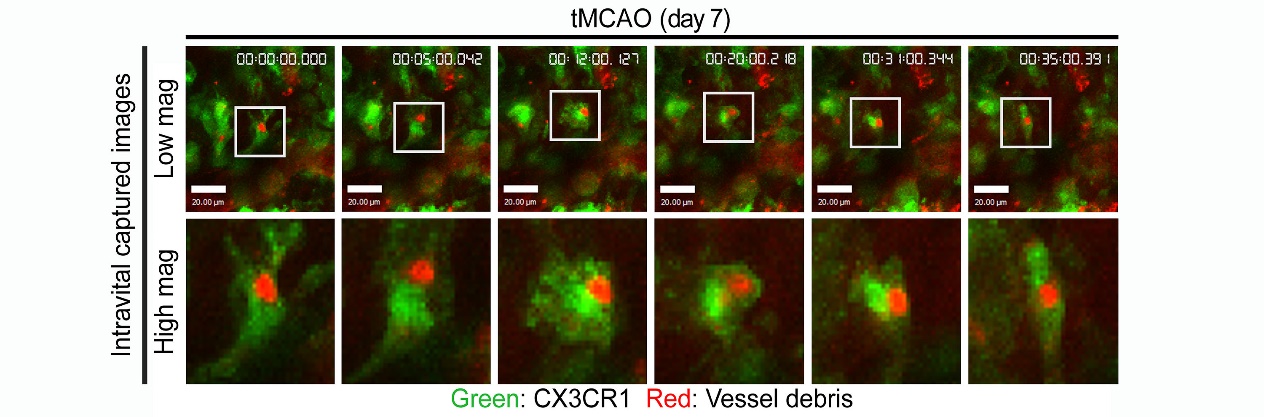


**Supplementary Figure 2**


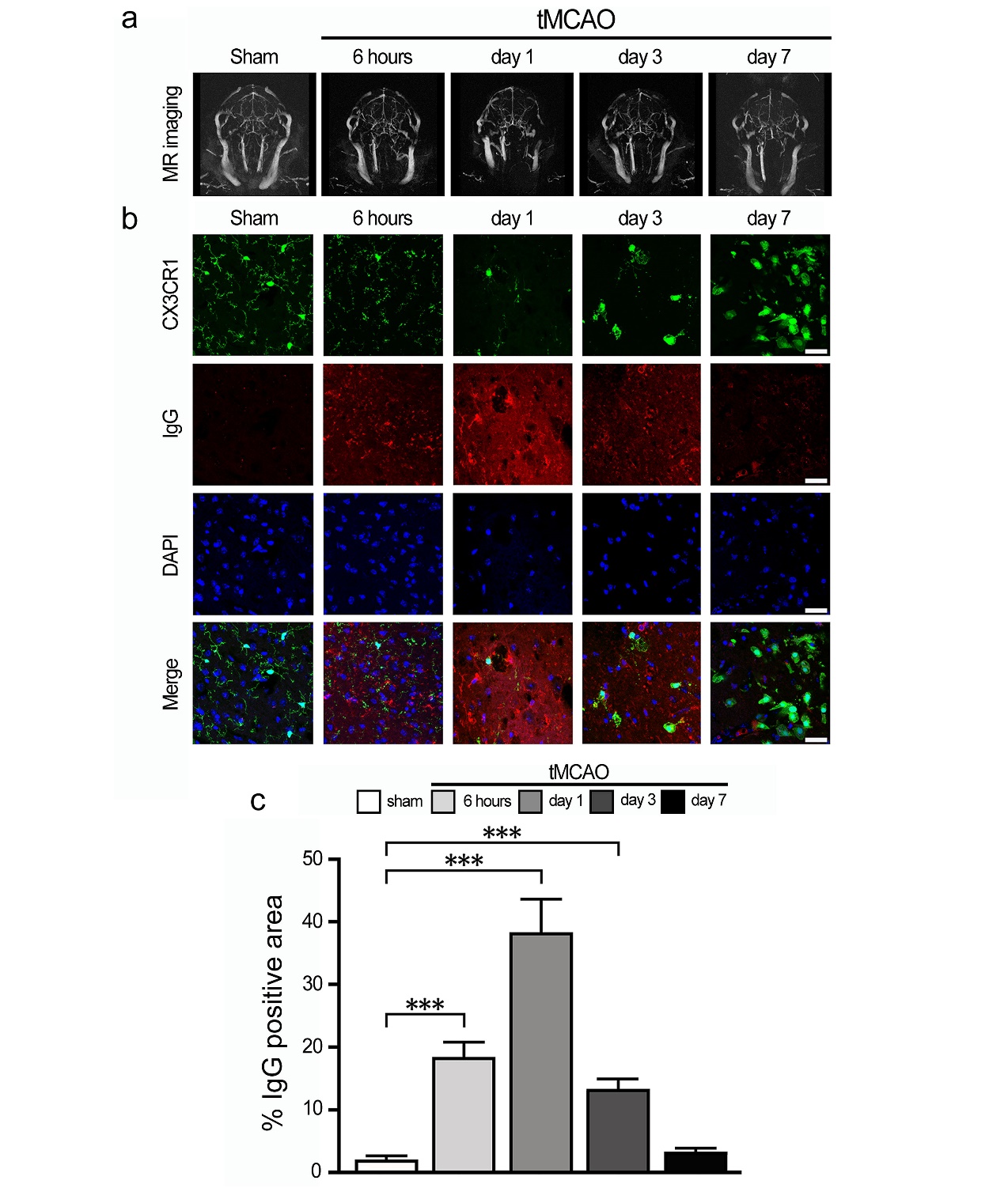

Supplement: Supplementary file 1 — (ZIP 1649 kb) [file 12975_2020_878_MOESM1_ESM.zip › Supple Figure_Translational stroke research/Supplementary Information_LJE.docx]
