# Supplementary figures and images for: Reparative System Arising from CCR2(+) Monocyte Conversion Attenuates Neuroinflammation Following Ischemic Stroke
Source: Transl Stroke Res. 2021 Jan 6;12(5):879–93. doi: 10.1007/s12975-020-00878-x (PMC8421302; doi:10.1007/s12975-020-00878-x)

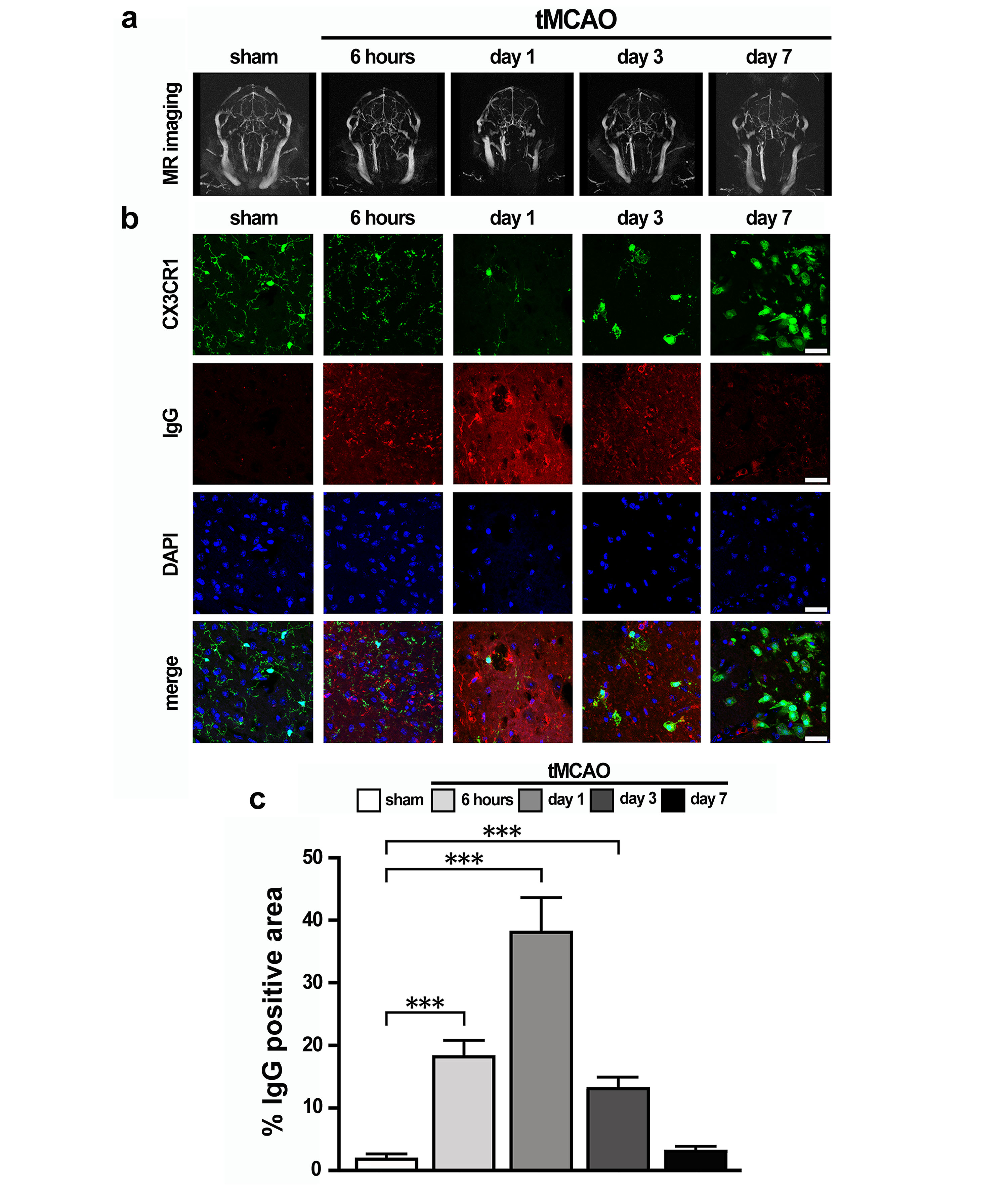

Supplement: Supplementary file 1 — (ZIP 1649 kb) [file 12975_2020_878_MOESM1_ESM.zip › Supple Figure_Translational stroke research/supple 1_20200723.jpg]

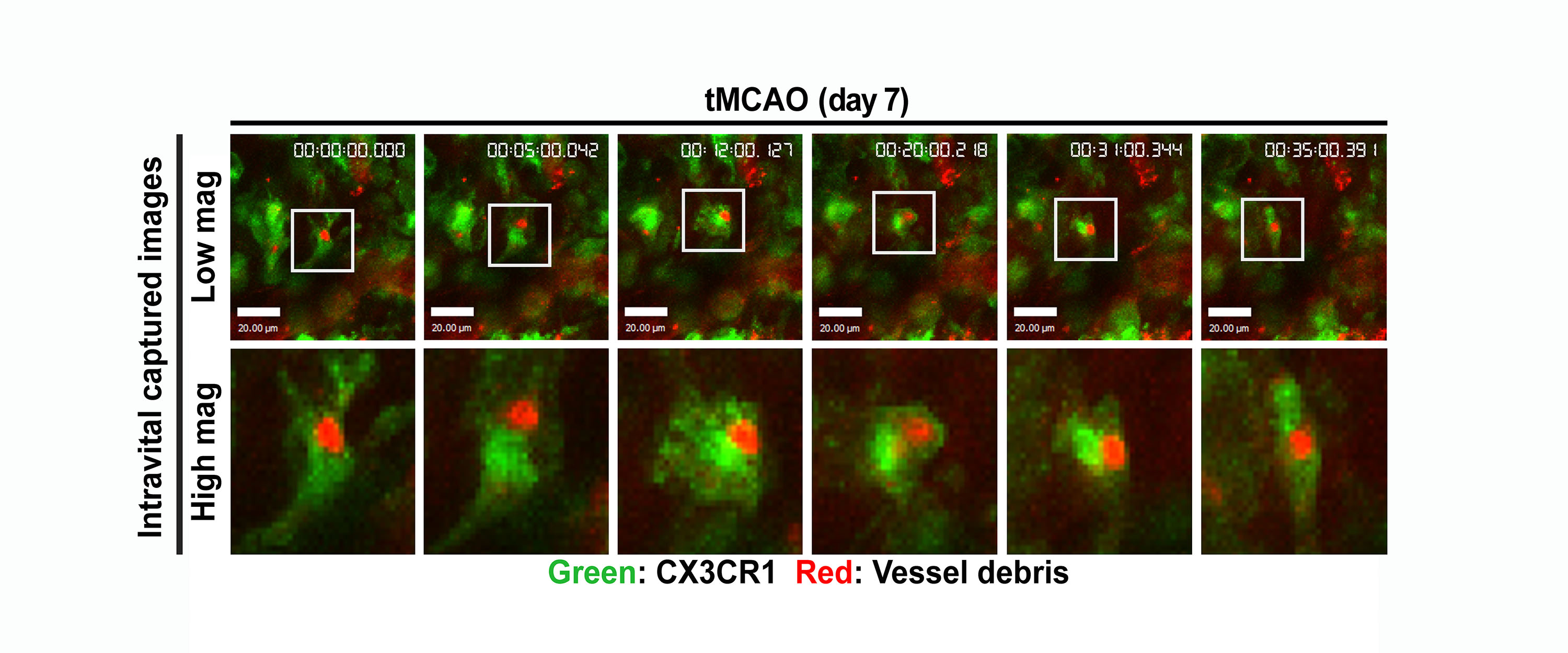

Supplement: Supplementary file 1 — (ZIP 1649 kb) [file 12975_2020_878_MOESM1_ESM.zip › Supple Figure_Translational stroke research/supple 2_20200723.jpg]
